# Novel aspects of glutamine synthetase (GS) regulation revealed by a detailed expression analysis of the entire GS gene family of *Medicago truncatula* under different physiological conditions

**DOI:** 10.1186/1471-2229-13-137

**Published:** 2013-09-21

**Authors:** Ana R Seabra, Liliana S Silva, Helena G Carvalho

**Affiliations:** 1 Instituto de Biologia Molecular e Celular da Universidade do Porto, Rua do Campo Alegre, 823, 4150-180 Porto, Portugal; 2 Current address: Max Planck Group for Fungal Biodiversity, Max Planck Institute for Plant Breeding Research, Carl-von-Linné-Weg 10, 50829 Köln, Germany

**Keywords:** Glutamine synthetase, Nitrogen fixation, Circadian rhythms, *Medicago truncatula*

## Abstract

**Background:**

Glutamine Synthetase (GS, EC 6.3.1.2) is a central enzyme in nitrogen metabolism, and a key component of nitrogen use efficiency (NUE) and plant yield and thus it is extremely important to understand how it is regulated in plants. *Medicago truncatula* provides an excellent model system to study GS, as it contain a very simple GS gene family comprising only four expressed genes, *MtGS1a* and *MtGS1b* encoding cytosolic polypeptides, and *MtGS2a* and *MtGS2b* encoding plastid-located enzymes. To identify new regulatory mechanisms controlling GS activity, we performed a detailed expression analysis of the entire GS gene family of *M. truncatula* in the major organs of the plant, over a time course of nodule or seed development and during a diurnal cycle.

**Results:**

Individual GS transcripts were quantified by qRT-PCR, and GS polypeptides and holoenzymes were evaluated by western blot and in-gel activity under native electrophoresis. These studies revealed that all four GS genes are differentially regulated in each organ of the plant, in a developmental manner, and identified new regulatory controls, which appear to be specific to certain metabolic contexts. Studies of the protein profiles showed that the GS polypeptides assemble into organ-specific protein complexes and suffer organ-specific post-translational modifications under defined physiological conditions. Our studies also reveal that GS expression and activity are modulated during a diurnal cycle. The biochemical properties of the four isoenzymes were determined and are discussed in relation to their function in the plant.

**Conclusions:**

This work provides a comprehensive overview of GS expression and regulation in the model legume *M. truncatula*, contributing to a better understanding of the specific function of individual isoenzymes and to the identification of novel organ-specific post-translational mechanisms of GS regulation. We demonstrate that the GS proteins are modified and/or integrated into protein-complexes that assemble into a specific composition in particular organs of the plant. Taken together, the results presented here open new avenues to explore the regulatory mechanisms controlling GS activity in plants, a subject of major importance due to the crucial importance of the enzyme for plant growth and productivity.

## Background

Nitrogen is an essential nutrient for plants and a major limiting factor in plant productivity. In plants, all inorganic nitrogen is first reduced to ammonium before it is incorporated into organic compounds. Ammonium is then assimilated by Glutamine Synthetase (GS) (EC.6.3.1.2) into glutamine, which provides nitrogen groups, directly or via glutamate, for virtually all nitrogenous cell compounds. The ammonium for GS activity is derived from the plant’s primary nitrogen sources (soil ammonium and nitrate, and atmospheric nitrogen in the case of legumes) as well as from a number of biochemical processes such as photorespiration, protein catabolism, deamination of amino acids and some specific biosynthetic reactions such as those involving methionine, isoleucine, phenylpropanoid and lignin [1].

Being the first enzyme of the nitrogen assimilatory pathway, GS is placed in a key position to play a regulatory role in nitrogen metabolism and plant productivity. Therefore a considerable amount of research has been dedicated to understand how GS is regulated and how it regulates nitrogen metabolism in plants (for reviews see [1-4]). Legumes have received special attention due to the key role that the enzyme plays in the assimilation of the ammonium produced by symbiotic nitrogen fixing rhizobia in root nodules. GS exists as a number of isoenzymes in higher plants, which are located in the cytosol (GS1) and in the plastids (GS2) and are encoded by a small multigene family. The subunits of cytosolic and plastid-located GS differ in molecular mass and can be readily separated by simple sodium dodecyl sulphate polyacrylamide gel electrophoresis (SDS-PAGE), GS1 polypeptides have a molecular mass of around 38–40 kDa whilst GS2 polypeptides are larger, ranging from 42 to 45 kDa [3]. The GS polypeptides have to assemble into multimeric complexes to be active. Based on extrapolations from the well-known structure of the bacterial enzyme [5], plant GS has long been considered an octameric enzyme. However, X-ray crystallography of maize ZmGS1a [6] and of *MtGS1a* from *M. truncatula*[7] demonstrated that the plant GS is a decameric enzyme. The protein is composed of two face-to-face pentameric rings, with the active sites formed at the interface between the N-terminal and C-terminal domains of two neighboring subunits within a pentameric ring, in a total of 10 active sites per GS decamer [6].

GS is a complex and highly regulated enzyme, which is controlled at many levels including transcription of the GS genes, mRNA stability, polypeptide synthesis and assembly, post-translational modifications and protein turnover [1,4]. The transcriptional regulation of GS has been best studied in several plant species. The GS genes show a complex pattern of expression, which is influenced by developmental and environmental cues, and their encoded isoenzymes play several, diverse but essential roles in nitrogen metabolism. Each of the GS genes appears to participate in different metabolic processes, based on where and how they are expressed. In root nodules of all legumes, one of the cytosolic genes is highly expressed in the infected cells, where it functions to assimilate the ammonia produced by nitrogen fixation [3,8]. The localization of a specific cytosolic GS isoenzyme in the vascular tissues has been reported for several species [9-12], suggesting an involvement of a particular GS isoenzyme in nitrogen transport. The plastid located GS is highly expressed in photosynthetic tissues, and its involvement in the reassimilation of the ammonium released by photorespiration has been unequivocally demonstrated using mutant plants [13]. The specific functions of the individual members of the cytosolic GS gene family are more difficult to define, and although plants have multiple genes encoding cytosolic GS isoenzymes, it has been traditionally more difficult to generate GS1 mutants. The first cytosolic GS knockout mutant was isolated in rice for the gene *OsGS1.1* and revealed that this isoenzyme has a remarkable effect in growth and grain filling [14]. Single and double mutants of maize GS1 knockout lines have also been isolated for the cytosolic GS genes *ZmGln1-3* and *ZmGln1-4,* and the mutant plants were also affected in growth and grain yield [15]. More recently, an Arabidopsis *Gln1.2* mutant was isolated and it appears that this gene is also involved in plant growth, but the effect was only clear when nitrate supply was high [16]. These three plant species have large GS1 gene families with 3 cytosolic GS genes in rice [17] and 5 in maize [18] and Arabidopsis [19]. For species containing a small number of cytosolic genes, as it is the case of *M truncatula*, with only two expressed cytosolic GS genes, generation of GS1 mutants is expected to be harder to achieve and thus the specific function of GS1 genes more difficult to access. The fact that the number of cytosolic genes is variable between different plant species makes it difficult to understand the specific function of a particular isoenzyme and relate it to the biochemical properties required for an adaptation to a certain physiological context. A complete picture of the factors controlling GS in a single plant is essential to clarify this aspect.

*Medicago truncatula* provides an excellent model system to study GS. This model legume contains one of the smallest plant GS gene families with only four expressed genes *MtGS1a* and *MtGS1b* encoding cytosolic polypeptides of 39 kDa and *MtGS2a* and *MtGS2b* encoding plastid located enzymes of 42 kDa [8,20]. We therefore chose this plant to try to obtain a holistic view of GS regulation in a single plant. The expression of the *M. truncatula* GS genes was previously characterized in several organs by northern blot, promoter-*gusA* fusions, *in situ* hybridization and immunolocalisation [9,21-23]. At the post-transcriptional level, *M. truncatula* GS isoenzymes were shown to be regulated by different mechanisms. GS2a is phosphorylated by Ca^2+^-dependent kinase(s) and phosphorylation allows interaction with 14-3-3 proteins, which then leads to selective proteolysis of the plastid located isoform, resulting in enzyme inactivation [24]. GS1a and GS1b are also targets for phosphorylation, yet by a Ca^2+^-independent kinase and its phosphorylation status is affected by light in leaves and by active nitrogen fixation in root nodules, which suggests a regulatory function related with circadian rhythms and nodule functioning [25]. In root nodules, it was also shown that GS1a is a molecular target of NO and suffers NO-mediated inactivation through tyrosine nitration. An increase in nodule GS nitration occurs in conditions in which nitrogen fixation is impaired and GS activity reduced (ineffective and nitrate-treated nodules) and it was suggested that this post-translational inactivation could be related to metabolite channelling, in order to boost the nodule antioxidant defences in response to NO [26].

Due to the crucial importance of GS for plant growth and productivity, it is extremely important to fully understand the mechanisms by which it is regulated in higher plants. The vital role of the enzyme and its involvement in many aspects of the complex matrix of nitrogen metabolism implies that GS has to be tightly controlled. In spite of the existence of considerable information on the different mechanisms of GS regulation from several plant species, clearly there are still many aspects to be discovered and many others that need to be clarified, especially regarding the mechanisms of posttranslational regulation, which are very poorly understood. In order to screen for new regulatory controls involved in the regulation of GS, we have carefully profiled GS transcripts, polypeptides and holoenzymes in the major organs of *M. truncatula*, during seed and root nodule development and during a diurnal cycle.

## Results

### Expression profile of the entire *M. truncatula* GS gene family in different organs of the plant

**Figure 1:**
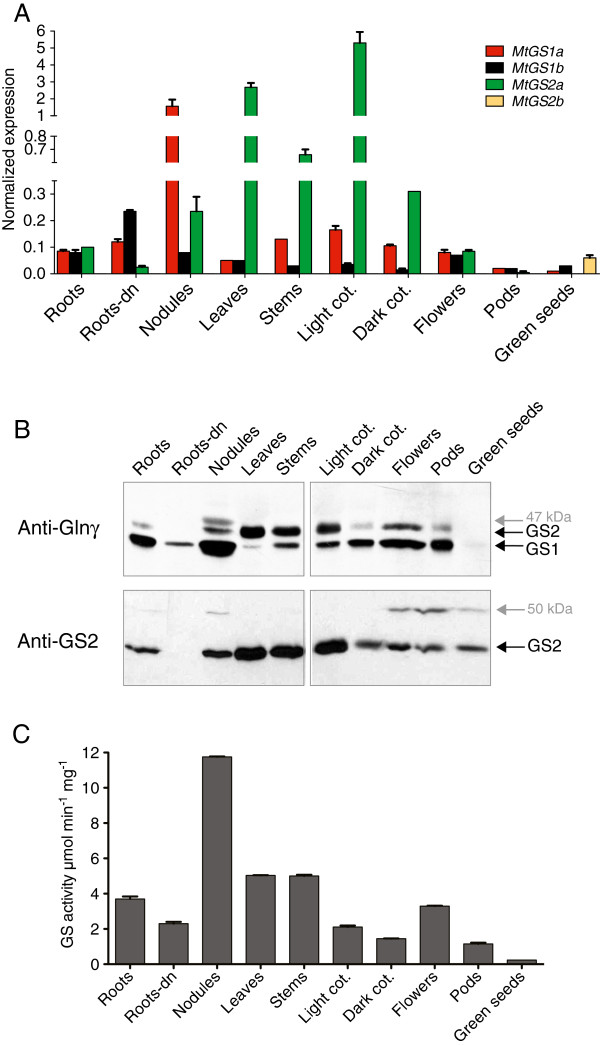
**Analysis of the expression of the entire *M. truncatula *GS gene family in several organs of the plant.** The organs analysed include roots, de-nodulated roots from N_2_-grown plants (Roots-DN), 14 day old nodules, leaves, stems, cotyledons from light (Light Cot) and from dark grown seedlings (Dark Cot), flowers, pods and green seeds. **A**. Quantification of *MtGS1a*, *MtGS1b*, *MtGS2a* and *MtGS2b* transcripts by qRT-PCR. GS expression was normalized to that of the housekeeping gene *MtElf1-α*. **B**. Western blot analysis of GS polypeptides using anti-Glnγ antibody [27] recognizing both the cytosolic (GS1) and plastid-located (GS2) polypeptides and a specific anti-GS2 antibody. Equal amount of protein were loaded on each lane, 30 μg for the anti-Glnγ antibody and 10 μg for the anti-GS2 antibody. **C**. Quantification of GS activity on soluble protein extracts. The results presented in this figure are representative of at least four biological replicates.

**Figure 2:**
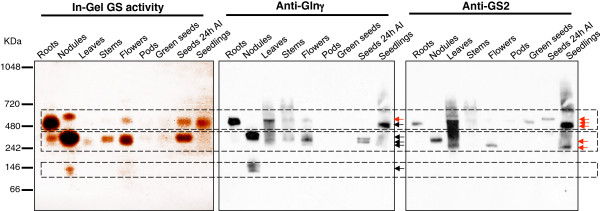
**Analysis of GS holoenzyme composition by Native polyacrylamide-gel electrophoresis in several organs of the plant.** The organs analysed include roots, nodules, leaves, stems, flowers, pods, green seeds, seeds 24 hours after water imbibition (24 HAI) and seedlings. Equal amounts of protein (30 μg) were loaded in each lane. In-gel GS activity is revealed by the brown staining. The native gel was Western blotted using the anti-GS2 and anti-Glnγ antibody [27]. The same membrane was first incubated with the specific anti-GS2 antibody, stripped and re-probed with the Anti-Glnγ antibody. Three major regions of GS activity are indicated in the gel. Red and dark arrows indicate GS2 or GS1 protein complexes, respectively. The images are representative of at least three independent experiments.

The expression of each member of the *M. truncatula* GS gene family was profiled in several organs of the plant. Transcript levels, polypeptide content and enzyme activity (Figure 1) were quantified and the GS holoenzyme complexes were analysed by native-gel electrophoresis (Figure 2) in the major organs of the plant. Individual GS gene transcripts were quantified by quantitative real time RT-PCR (qRT-PCR) in roots grown on ammonium nitrate, de-nodulated roots, root nodules, leaves, stems, light or dark grown cotyledons, flowers, pods and seeds (Figure 1A). This analysis confirmed the previously reported specific expression of *MtGS2b* in the seeds [20] and quantified the differential expression of *MtGS1a*, *MtGS1b* and *MtGS2a* in defined organs of the plant. It is noteworthy the significantly lower expression of *MtGS1b*, relatively to *MtGS1a* and *MtGS2a*, in most organs and conditions tested, being expressed at modestly higher levels in roots and flowers. *MtGS1a,* previously shown to be the *M. truncatula* nodule enhanced GS gene, responsible for the assimilation of nitrogen fixed ammonia, is 14 fold overexpressed in root nodules in comparison to roots. *MtGS2a* mRNA was found to accumulate very strongly in photosynthetic organs, showing 30 fold increased expression in leaves and 50 fold increased expression in green cotyledons in relation to roots (Figure 1A).

To investigate whether the variations in GS transcripts correlate with the polypeptide content, total soluble proteins were extracted from each organ and analysed by western blot using two different GS antibodies, one specific for the plastid located GS2 (anti-GS2), and another recognizing both cytosolic and plastid-located polypeptides (anti-glnγ, [27] (Figure 1B). Cytosolic (GS1) polypeptides are highly abundant in roots, nodules, flowers, pods and dark cotyledons, whereas the plastid-located GS2 is mainly present in photosynthetic organs such as leaves, stems, and cotyledons of seedlings exposed to light (Figure 1B). It is noteworthy that in roots, the GS polypeptide content appears to be dependent on the presence of inorganic nitrogen, as it is greatly reduced in de-nodulated roots in comparison to roots of plants grown on NH_4_NO_3_ (Figure 1B). The results also indicate that the accumulation of GS1 polypeptides is negatively affected by light in cotyledons, contrasting with GS2 for which the polypeptide content is increased in light grown cotyledons (Figure 1B).

One striking observation from this simple western blot analysis is the detection of additional GS polypeptides of higher molecular mass in specific organs of the plant, indicating the occurrence of post-translational modifications. A GS polypeptide of around 47 kDa is detected exclusively in root nodules (Figure 1B) and, as this polypeptide is not recognized by the specific anti-GS2 antibody, it likely represents a GS1 post-translational modification occurring specifically in root nodules. An additional GS polypeptide of around 50 kDa was detected in nodules, but also in flowers, pods and green seeds. This polypeptide is specifically recognized by the anti-GS2 antibody and probably represents a GS2 post-translational modification occurring in these four organs (Figure 1B).

Total GS activity was generally consistent with the overall GS polypeptide abundance in each organ (Figure 1C). Root nodules showed the highest GS activity of all organs, correlating with the highest GS1 polypeptide content. However, the GS polypeptide abundance does not always correlate with GS activity levels, for example in de-nodulated roots, GS activity was comparable to that observed in cotyledons but the GS polypeptide content is greatly reduced. Taken together these results indicate new regulatory controls operating to modulate GS activity in different organs of the plant.

### Analysis of GS holoenzyme composition in different organs

To analyse GS holoenzymes and protein complexes present in each organ of the plant, we performed native-PAGE of protein extracts from different organs of *M. truncatula*, followed by activity staining or immunoblot analysis using the two anti-GS antibodies (Figure 2). Multiple brown-stained proteins indicate the existence of several protein complexes with GS activity, which were roughly distributed into three classes according to their M_r_ (Figure 2). Since GS is a decameric enzyme, the native molecular mass of cytosolic GS holoenzymes is expected to be around 390 kDa, whereas the plastid located complexes should assemble as a holoenzyme of 420 kDa, and thus both GS1 and GS2 are expected to migrate between molecular markers of 480 and 242 kDa. Indeed, proteins with GS activity of compatible size could be detected in all organs analysed, but slow-migrating proteins with GS activity were also observed in extracts from roots, nodules, flowers, seeds and seedlings, and a GS active fast–migrating protein was detected exclusively in nodules.

To investigate which of the native complexes correspond to cytosolic or plastid located enzymes, the native gels were subjected to western blot analysis. The membranes were first probed with the anti-GS2 antibody and then re-probed with anti-Glnγ antibody (Figure 2). This analysis identified at least five differently migrating GS2 immuno-reactive proteins (red arrows), and five major proteins related to GS1 (black arrows). It should be noted that the electrophoretic mobility of the isoenzymes on native gels is dependent on the net charge, size and the conformational arrangement of the subunits and GS2 migrates faster than GS1 in our gel system (Figure 2), what was confirmed by an analysis of purified recombinant GS1a and GS2a proteins (data not shown).

Slow-migrating GS2 holoenzymes of around 480 kDa were mostly abundant in roots, leaves, green seeds, seeds following imbibition and in seedlings. Regarding GS1, a protein with the expected native Mr of 390 kDa was abundantly present in nodules but also found in leaves, stems, flowers, seeds and seedlings. An additional slow-migrating complex was detected in roots, leaves, stems, flowers, and seedlings by the anti-Glnγ antibody, which cross-reacts with both GS1 and GS2. In leaves and seedlings this slow migrating protein complex likely corresponds to GS2 because it is strongly recognized by the specific GS2 antibody. The existence of multiple GS species in different organs of the plant indicates the occurrence of protein complexes and/or post-translation modifications in an organ-specific manner, a finding of particular interest.

### Expression profile of GS during nodule development

**Figure 3:**
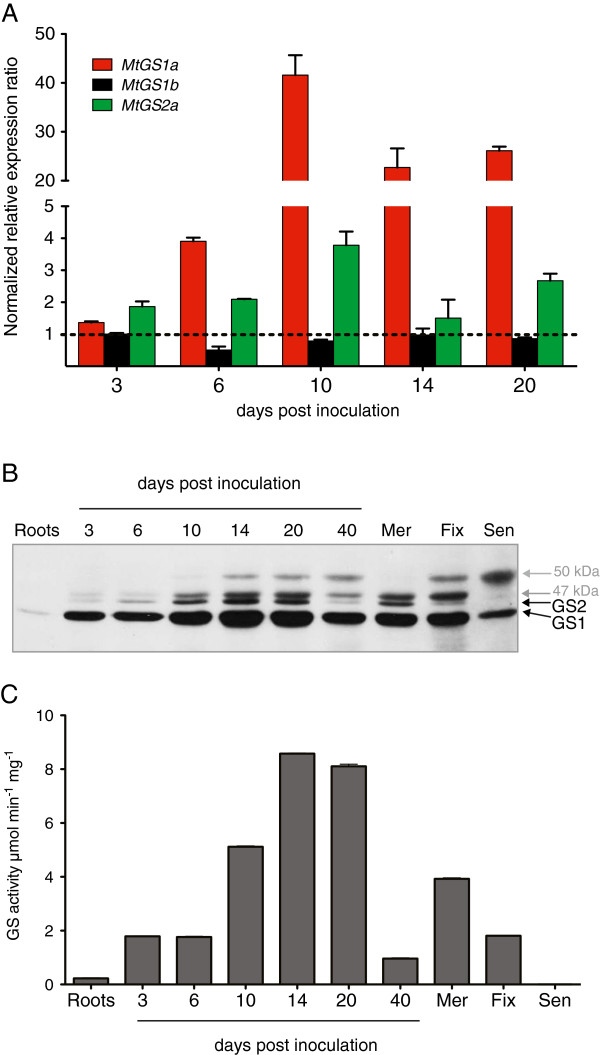
**Analysis of the expression of the entire *M. truncatula *GS gene family during nodule development.** Nodules were collected at 0, 3, 6, 10, 14, 20 and 40 days after infection. **A**. Quantification of *MtGS1a*, *MtGS1b*, *MtGS2a* and *MtGS2b* transcripts by qRT-PCR. GS transcript abundance was normalized to that of the housekeeping gene *MtElf1-α*. **B**. Western blot analysis of GS polypeptides using anti-Glnγ antibody recognizing both the cytosolic (GS1) and plastid-located (GS2) polypeptides [27]. 40 day old nodules were dissected into three parts, enriched in meristematic zone (Mer), nitrogen fixation zone (Fix) and senescence zone (Sen). Equal amount of protein (30 μg) were loaded on each lane. **C**. Quantification of GS activity on soluble protein extracts. Results are representative of at least 3 biological replicates.

To try to identify specific regulatory controls occurring during nodule development, we followed transcript levels, polypeptide content and GS activity over a time course of nodule development, in comparison to roots, collected before rhizobial inoculation. Surprisingly, the expression of both *MtGS1a* and *MtGS2a* was approximately 2-fold up regulated very early in symbiosis (3 dpi). After the onset of nitrogen fixation (10 dpi), the expression of *MtGS1a* was strongly enhanced to approximately 40-fold whilst *MtGS2a* transcripts accumulate to moderate levels. Thereafter (14 and 20 dpi), the expression of *MtGS1a* and *MtGS2a* was fairly sustained. *MtGS1b* was expressed at very low levels at all times. The GS polypeptide content follows a rapid increase during nodulation, reaching a constant level at 14 dpi, with GS1 being considerably more abundant than GS2 (Figure 3B). The increased GS polypeptide content is accompanied by a corresponding increase in total GS activity over the time course of nodule development (Figure 3C).

For a more detailed characterization of GS distribution in the three main histological zones of the nodule, 40 dpi root nodules were roughly separated into three parts, meristem (Mer), fixation zone (Fix) and senescent zone (Sen). The decrease in polypeptide content and total GS activity observed at 40 dpi, probably reflects the existence of a considerably large senescent zone in older nodules (Figure 3C). Due to technical difficulties in isolating meristems, GS activity in the meristem fraction was found to be higher than in the fixation zone, most certainly due to the inclusion of interzone II-III, where GS is particularly active [21]. In spite of these technical limitations, this analysis provided relevant information concerning both the occurrence of post-translational modifications and the differential expression of GS2 and GS1 in the different zones of the nodule. Interestingly, the 50 kDa GS2 polypeptide, described in the previous section, abundantly accumulates in the senescent zone of the nodule and is not detected in the meristem-enriched fraction (Figure 3B) suggesting a correlation of this post-translational modification with senescence. Conversely, the putative GS1 47 kDa post-translationally modified polypeptide was absent in the senescent zone and abundantly present in the fixation zone (Figure 3B) indicating a relationship with active N_2_-fixation.

### Expression profile of GS during seed development

**Figure 4:**
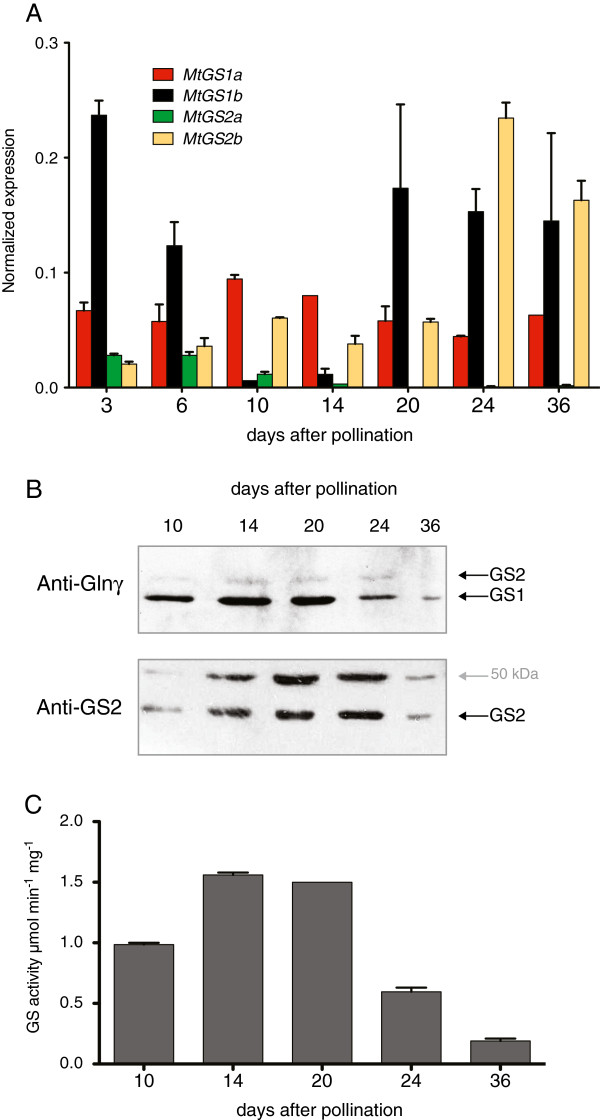
**Analysis of the expression of the entire *M. truncatula *GS gene family during seed development.** Seeds were collected at 3, 6, 10, 14, 20, 24 and 36 days after pollination. **A**. Quantification of *MtGS1a*, *MtGS1b*, *MtGS2a* and *MtGS2b* transcripts by qRT-PCR. GS transcript abundance was normalized to that of the housekeeping gene *MtElf1-α*. **B**. Western blot analysis of GS polypeptides using either an anti-Glnγ antibody recognizing both the cytosolic (GS1) and plastid-located (GS2) polypeptides [27] or a specific anti-GS2 antibody. Equal amount of protein (30 μg) were loaded on each lane. **C**. Quantification of GS activity on soluble protein extracts. Results are representative of three biological replicates.

The expression of the GS gene family was also analysed during seed development by comparing transcript levels, polypeptide content and enzyme activity (Figure 4). All four GS genes are expressed in the seeds, but are differentially expressed over the time course of seed development. *MtGS2a* is expressed at moderate levels in the early stages of development, and it ceases to be expressed 14 days after pollination, whereas the seed specific gene *MtGS2b*, is expressed all through the time course of seed development, but is most strongly expressed at the late stages of seed filling (24 to 36 DAP). Regarding the cytosolic genes, *MtGS1a* is expressed at relatively constant levels during whole seed development, whereas the expression of *MtGS1b* is higher at early seed developmental stages, decreases to almost undetectable levels at 10 to 14 DAP and increases thereafter (20 to 36 DAP). Interestingly, cytosolic GS polypeptide abundance do not exactly follow the corresponding transcript levels, specially at later stages of development (24 to 36 DAP), when both *MtGS1a* and *MtGS1b* are well expressed, but a decrease in GS1 polypeptide content is clearly observed. In contrast, GS2 polypeptide abundance was higher at middle stages of seed development (14 to 24 DAP) and interestingly, also the 50 kDa GS2 immunoreactive polypeptide, which follows the pattern of the mature GS2 polypeptide accumulation. GS activity in the seeds is higher at the stages of seed filling (14 to 20 DAP), decreasing significantly, at the later stages of seed development.

### Expression profile of GS during a diurnal cycle

**Figure 5:**
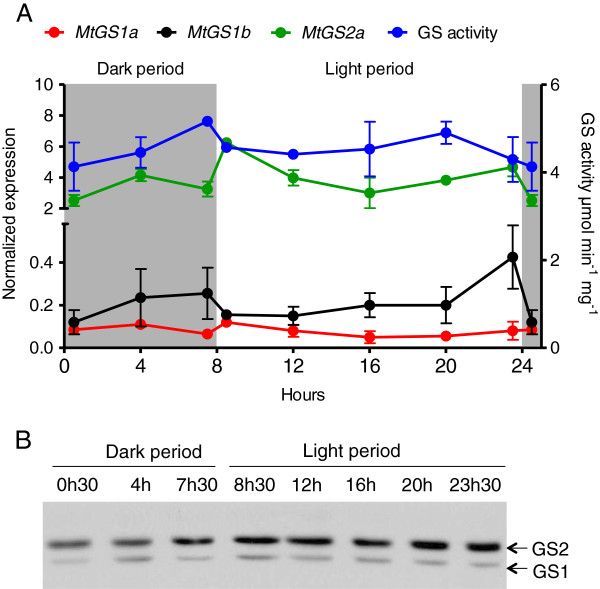
**Analysis of the expression of *MtGS1a*, *MtGS1b *and *MtGS2a *during a diurnal cycle.** Leaf samples were collected 30 minutes before or after the transition day/night and night/day and every 4 hours over a period of 24 hours. **A**. The graphic represents the quantification of *MtGS1a*, *MtGS1b* and *MtGS2a* transcripts by qRT-PCR, normalized to the housekeeping gene *MtElf1-α,* and the determination of total GS activity in the leaves. **B**. Western blot analysis using the Anti-Glnγ antibody. Equal amount of protein (30 μg) was loaded on the gel. Results are representative of two independent experiments, each using three biological replicates.

To evaluate whether the expression of GS is influenced by circadian rhythms, GS transcripts, activity and polypeptide content were analysed during a diurnal cycle in leaves of *M. truncatula*. Total leaf GS activity was found to oscillate during the diurnal cycle, but not extensively, and surprisingly the activity was highest 30 minutes before illumination, decreasing during the first 30 minutes of light (Figure 5A). Since GS2a is the main leaf GS isoform, the observed variations in GS activity should reflect variations in GS2 activity. However, the decrease in GS activity detected at the beginning of the light period was accompanied by an increase in the transcription of *MtGS2a* (Figure 5A) and also by an increase in GS2 polypeptide abundance (Figure 5B), strongly suggesting a post-translational inactivation of GS2 at the beginning of light period. During the dark, *MtGS2a* transcripts as well as the GS2 polypeptide content remained essentially constant. Both *MtGS1a* and *MtGS1b* GS transcripts, and cytosolic polypeptides were maintained at relatively low levels during the diurnal cycle (Figure 5B).

### Comparison of the kinetic properties of the four *M. truncatula* GS isoenzymes

To further characterize the entire *M. truncatula* GS protein family, we compared the catalytical properties of the four GS isoenzymes. The plant proteins were produced in *Escherichia coli*, with a his-tag and purified to homogeneity. An examination of the kinetic properties of purified GS1a, GS1b, GS2a and GS2b revealed some differences among them. The first remarkable difference relates to the specific activity of GS2b, which display a surprisingly high synthetase activity (GSs) when compared to the other three isoenzymes and an unusual low transferase activity (GSt) (Table 1). In contrast, GS1a displayed the highest GSt activity (413 μmol.min^-1^.mg^-1^). The transferase to synthetase activity ratios (GSt:GSs) were 87, 145, 56 and 1.2 for GS1a, GS1b, GS2a and GS2b, respectively (Table 1). The pH optimum for the cytosolic isoenzymes (GS1a and GS1b) was around 7.0 whereas the pH optimum for the two plastid-located isoenzymes (GS2a and GS2b) was slightly higher (pH 7.5).

**Table 1:** **Kinetic properties of the four *M. truncatula *GS isoenzymes**

	**GS1a**	**GS1b**	**GS2a**	**GS2b**
*K*_*m*_ Glu (mM)	2.76 ± 0.22	1.56 ± 0.13	4.47 ± 0.30	6.79 ± 0.58
*K*_*m*_ ATP mM	2.34 ± 0.18	1.67 ± 0.17	1.73 ± 0.14	4.76 ± 0.61
*K*_*0.5*_ Hydroxylamine (mM)	0.90 ± 0.09	0.83 ± 0.08	0.97 ± 0.10	2.09 ± 0.27
*h* Hydroxylamine	1.97 ± 0.20	1.73 ± 0.16	1.73 ± 0.17	1.57 ± 0.14
GSs (μmol min^-1^ mg^-1^)	4.78	2.04	4.46	9.58
GSt (μmol min^-1^ mg^-1^)	413	296	251	12
Ratio GSt:GSs	87	145	56	1.2
pH optimum	7.0	7.0	7.5	7.5

The affinity of each GS isoenzyme for the substrates was estimated using the synthetase reaction and 10 μg (GS1a; GS2a and GS2b) or 20 μg (GS1b) of purified protein per assay. Values were automatically calculated by Prism5® software (Graphpad software Inc.) by non-linear least squares regression method assuming either a Michaelis-Menten kinetics (hyperbolic) or an allosteric sigmoidal kinetics. *K*_*m*_ – Michaelis-Menten constant; *K*_*0.5*_ – Half-saturation constant; *h* – hill coefficient.

**Figure 6:**
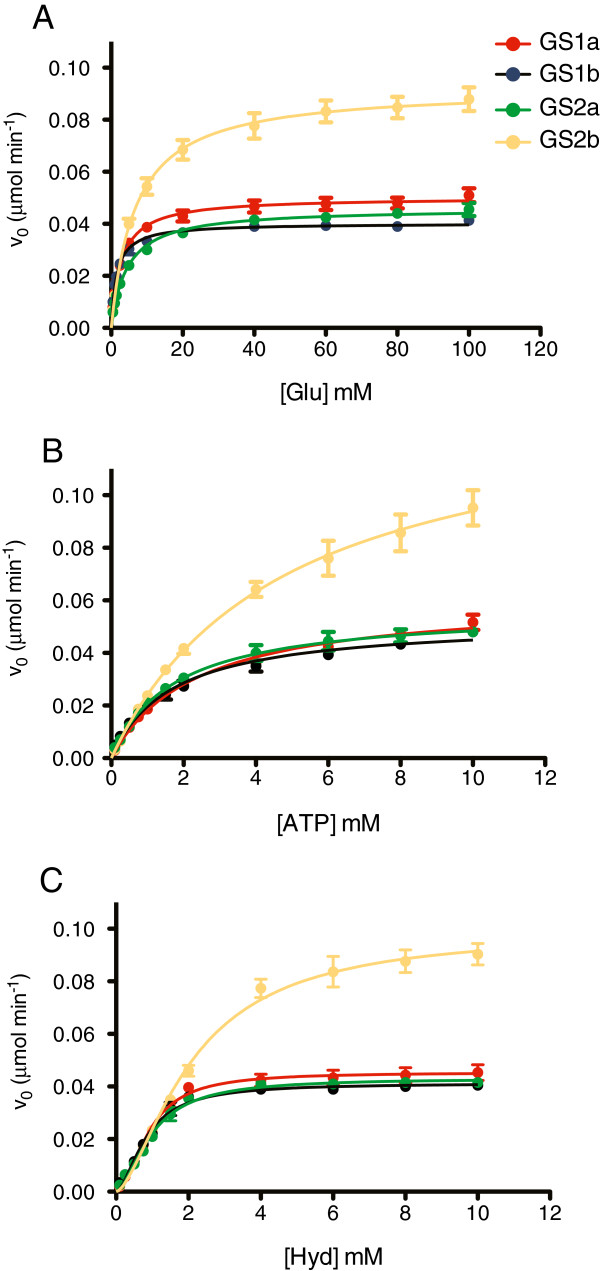
**Kinetics of *MtGS1a*, *MtGS1b *and *MtGS2a*.** Dependence of *v*_*o*_ on glutamate **(A)**, ATP **(B)** and hydroxylamine **(C)** concentration. The “initial rate” (*v*_*0*_) of the synthetase GS reaction, catalyzed by the purified His-tagged proteins is expressed as μmol of γ-glutamyl hydroxamate produced per minute.

The dependence of *v*_*0*_ on the concentration of each substrate was evaluated for the different isoenzymes (Figure 6). To estimate the “initial rate” (*v*_*0*_) of the GSs reaction, the production of hydroxamate was measured at several time points during 15 minutes and exhibited a linear increase during the first 3 minutes for all four isoenzymes (data not shown). The four isoenzymes followed a normal Michaelis-Menten kinetics with respect to L-glutamate and ATP, as evidenced by the hyperbolic curves observed in Figure 6A and B and thus the Michaelis-Menten constant (*K*_*m*_) was determined for glutamate and ATP and is presented in Table 1. Since the progression of the GSs reaction with respect to hydroxylamine follows a sigmoidal curve (Figure 6C), the kinetic parameters calculated were the half-saturation constant (*K*_*0.5*_) and the Hill coefficient (*h*). These kinetic analysis showed that GS1b exhibit a higher affinity for all three substrates, whereas GS2b exhibit the lowest affinity (Table 1). The calculated *h* values for hydroxylamine are close to 2 indicating positive cooperativity kinetic for all four isoenzymes.

## Discussion

To screen for new regulatory controls and obtain insights into the specific physiological functions of the individual *M. truncatula* GS isoenzymes, we performed a detailed expression analysis of the entire GS gene family of *M. truncatula* under several physiological conditions and compared the kinetics of the four isoenzymes. These analysis revealed new interesting aspects related to the differential expression of the individual members of the *M. truncatula* GS gene family. Clearly *MtGS1a* and *MtGS2a* encode the major GS isoenzymes in *M. truncatula.* The two genes show the highest expression in almost all organs of the plant, with *MtGS1a* being particularly highly expressed in root nodules and *MtGS2a* in photosynthetic tissues. This is in general agreement with previous qualitative expression analysis of three of the *M. truncatula* GS genes, by northern blot [23] and *MtGS* promoter-*gusA* fusions [9,21]. However, the present quantitative analysis revealed that *MtGS1b* is expressed at relatively low levels in almost all organs of the plant and revealed subtle differences in the expression of the individual GS genes during development and diurnal cycles. During nodule development, *MtGS1a* and *MtGS2a* constitute the main up regulated GS genes, as previously shown [21,22]. *MtGS2a* is expressed at relatively low levels over the course of nodule development*,* whereas *MtGS1a* is induced to very high levels (around 20 fold in relation to roots). Contrariwise, the expression of *MtGS1b* appears to decrease in root nodules in relation to roots. It has been previously shown that GS1a is located at the infected cells in root nodules, where it functions to assimilate the ammonia derived from bacterial nitrogen fixation [21]. The kinetic properties of GS1a indicate a lower affinity of this isoenzyme for the three substrates, when compared to its cytosolic counterpart GS1b, but a higher specific synthetase activity (GSs), which reflect an enzyme more suited to a metabolic environment where ammonium is expected to be highly abundant, and needs to be quickly assimilated, in order to prevent toxic effects. The kinetic properties, together with the low and constitutive expression of *MtGS1b* in all organs of the plant are compatible with a housekeeping function of the encoded isoenzyme.

Regarding seed development, the present quantitative analysis confirmed the previously reported seed specific expression of *MtGS2b*[20] and revealed that all four genes are differentially expressed during seed development. The other plastid located GS gene, *MtGS2a*, is poorly expressed at all stages and is not expressed at the late stages of seed filling, a time when *MtGS2b* and *MtGS1b* are the major expressed GS genes. This has led to the proposal that the isoenzyme GS2b is involved in supporting storage compound biosynthesis during seed filling [20]. At the catalytic level, GS2b shows some unusual kinetic properties, with a low affinity for all substrates, an unusually high GS synthetase activity and a surprisingly low GS transferase activity. Since the enzyme is the result of a recent gene duplication event and is exclusively expressed in the seeds [20], these properties suggest a kinetic advantage adapted to the unique metabolism of legume seed plastids.

The metabolic fluctuations that plants suffer during a day imply that the plant enzymes have to be subjected to diurnal regulation. However there are very few reports on the diurnal regulation of GS expression, and most of them have been devoted to roots [28-32]. Our results reveal that the circadian clock influences the expression of GS genes in *M. truncatula* at the transcriptional, translational and post-translational levels. *MtGS2a* mRNA accumulates during the start of the light period and this is accompanied by a slight increase in polypeptide abundance, however total GS activity in the leaves decreases within the first 30 minutes of light. Similarly, it was reported that *ZmGln2* mRNA peaks early in the light period in maize leaves [32]. The main function of the plastid-located GS in leaves of C_3_ plants is the assimilation of ammonium resulting from photorespiration [13]. Thus it would be expected that *MtGS2a* is more highly expressed during the day to prevent ammonium accumulation. However, in Arabidopsis it does not seem that photorespiratory ammonium directly regulates GS2 expression [33]. And in fact, it appears that photosynthetic sugars can serve as intermediates for the regulation of GS2. It has been shown that GS2 is positively modulated by CO_2_ assimilation in leaves of sunflower, but the process is mediated through formed sugars and not by photorespiration [34]. As it is well known that sugars undergo marked diurnal changes in leaves [35] it is tempting to consider that the diurnal pattern of GS2 expression is responsive to these sugar fluctuations. Our studies indicate that the enzyme is inactivated post-translationally at the beginning of the light period. Further studies should address whether sugar, direct light, or signals related to internal rhythms influence the diurnal oscillation of GS activity.

The analysis of in-gel GS activity coupled to western blot of native gels revealed that GS polypeptides assemble into organ-specific protein complexes. In addition to GS active complexes of the expected Mr (390 kDa for decameric GS1 and 420 kDa for decameric GS2), additional slow migrating and fast migrating protein complexes were detected. This heterogeneity suggests that the proteins are modified and/or integrated into protein-complexes that assemble into a specific composition in particular organs. Especially interesting is the fast migrating active GS1 protein detected in nodules. Since GS is composed of two pentameric rings with an estimated M_r_ of 195 kDa [6,7], this slow migrating protein is likely to correspond to a dissociated pentameric ring. Since the active site of GS is formed at the interface of two neighbour subunits within each ring [6], it is conceivable that the dissociated rings are catalytically active. The finding that this complex is detected only in nodules suggests that dissociation is increased in this organ. GS dissociated rings were also reported to be active in roots of *Beta vulgaris*[36], but the physiological meaning for the existence of dissociated pentameric rings remains to be elucidated.

Interestingly, in addition to the detection of organ specific GS holoenzyme complexes, we also detected organ specific post-translational modifications of both GS1 and GS2 polypeptides. A higher molecular weight GS1 polypeptide with an apparent Mr of 47 kDa was specifically detected in root nodules and found to accumulate during nodule development. The finding that the accumulation of this polypeptide is associated with the nodule nitrogen fixation zone is particularly stimulating as it indicates a nodule specific post-translational regulation related with active nitrogen fixation. Regarding GS2, a polypeptide of 50 kDa was detected in nodules, flowers, pods and seeds, reflecting a specific GS2 post-translational modification common in these four organs. Remarkably, in root nodules, this GS2 polypeptide of higher molecular weight is clearly associated with the senescent zone. The apparent Mr of these polypeptides 47 kDa for GS1 and 50 kDa for GS2 implies a covalently bound additional peptide matching the size of ubiquitin or ubiquitin-like proteins (approximately 8 kDa) to the regular GS polypeptides of 39 and 42 kDa for GS1 and GS2, respectively. It is thus tempting to speculate that these polypeptides could result from binding of one molecule of ubiquitin, SUMO or other ubiquitin-like protein. Monoubiquitination is recognized as a reversible post-translational modification involved in the regulation of diverse processes such as membrane transport, transcription and signal transduction [37,38]. Interestingly, it has recently been shown that another important metabolic enzyme, PEPC, is regulated by monoubiquitination in germinating seeds of castor bean [39]. Also, GS2 has been identified as a potential SUMO substrate by two-hybrid in Arabidopsis, but the physiological meaning of this process was not investigated [40]. Although at the present stage any consideration regarding the physiological implications of GS modification by ubiquitin like proteins can only remain highly speculative, the accumulation of the 50 kDa GS2 polypeptide in the senescence zone of root nodules suggests that the post-translational modification could operate to target the protein for degradation, whereas the association of the 47 kDa GS1 polypeptide with the nitrogen fixation zone indicates a regulatory function related to active nitrogen fixation.

## Conclusions

In conclusion, the results presented here extend our knowledge on the expression and regulation of the GS family in *M. truncatula* and contributes to a better understanding of the specific function of individual isoenzymes. These studies revealed that all four *M. truncatula* GS genes are differentially regulated in each organ of the plant, in a developmental manner, and identified new regulatory controls, which appear to be specific to certain metabolic contexts. It is shown that GS expression and activity are modulated during a diurnal cycle, an aspect that should be thoroughly investigated in the future, as it is important to understand how GS expression is adjusted to the diurnal metabolic rhythms that tightly link carbon and nitrogen assimilation. Finally, this study opens new interesting questions regarding post-translational modifications, such as the association of GS with other proteins to form organ-specific protein complexes and the possible regulation of both GS1 and GS2 by ubiquitin or ubiquitin-like proteins. Further work will be required to characterize the proteins binding each individual GS isoenzyme and understand the molecular and physiological consequences of those post-translational modifications for nitrogen metabolism, nevertheless, the results presented here are relevant as they open new avenues to explore the regulatory mechanisms controlling GS activity in plants, a subject of major importance to current agricultural issues due to the crucial importance of the enzyme for crop growth and productivity.

## Methods

### Plant material and growth conditions

*Medicago truncatula* Gaertn. (cv. Jemalong J5) was grown in aeroponic conditions under 16 h light (22°C)/8 h dark (19°C) cycles and under a light intensity of 150–200 μmol m^-2^ s^-1^, in the growth medium described by [41]. For nodule induction, the growth medium was replaced with fresh medium lacking a nitrogen source three days before inoculation with *Sinorhizobium meliloti* strain Rm1021 pXLGD4 RCR 2011(GMI 151). Root nodules were separated from roots and frozen separately. Nodules were harvested at 0, 3, 6, 10, 14, 20 and 40 dpi. Nodules collected at 40 dpi were hand sectioned transversally into 3 parts to obtain samples enriched in meristem, nitrogen fixation and senescence zones. Flowers, pods and seeds, were collected from plants grown on soil and fertilized once a week. Cotyledons were collected from 5 day-old seedlings either kept in the dark or exposed to light. For the study of circadian rhythms, plants were grown aeroponically as described above and leaves were collected at several time points during 24 hours: 30 minutes before or after the transition day/night and night/day and every 4 hours. All plant material was immediately frozen in liquid nitrogen and stored at – 80°C.

### RNA extraction and quantitative real time PCR

Total RNA was isolated from 100 mg of plant tissue, using the RNeasy Plant mini Kit (Qiagen) according to the manufacturer instructions, with an extra step of on-column DNAse digestion. Total RNA was quantified using a Nanodrop spectrophotometer (Thermo scientific) and its integrity was verified in gel. Each sample comprises biological material pooled from 5 different plants. Total RNA (2.0 μg) was reverse transcribed using Superscript-RT™ III (Invitrogen, Life Science) and random hexamers according to the manufacturer’s instructions. Quantitative RT-PCR was performed using an iCycler Thermal cycler (Bio-rad) detection system using iQ™ SYBR® Green Supermix (Bio-rad). Three technical replicates were performed for each primer pair and sample combination, in 20 μL reaction volume including 25 ng of cDNA and 5 pmol of each primer: 5′GTGTTCTTCTTCTTCCTTCAC3′ and 5′GGTGTAAACATCACAAATCAC3′ for *MtGS1a*; 5′ATAAGCCACCACGCTACTTC3′ and 5′AACCATAACAAGGACTCAGATC3′ for *MtGS1b*; 5′TCACTTGAACCCATTTCCTAAG3′ and 5′CCAGAGTTGACTGCCATTAC3′ for *MtGS2a*; 5′ATCTGGTGTCTGACACAGCAAAAC3′ and 5′GCCAGAGTTGATTGCCATTGC3′for *MtGS2b*, 5′CCACCAACCTTGACTGGTAC3′ and 5′CCACGCTTGAGATCCTTCAC3′ for *MtElf1-α.* The amplification conditions were as follows: initial denaturation (95°C for 3 min) followed by 40 cycles of amplification and quantification (95°C for 10s, 54°C for 30s and 72°C for 30s with a single fluorescence measurement) and melting curve generation (55°C to 95°C with one fluorescence read every 0.5°C). Calculation of the cycle threshold (C_t_) and primer efficiency was performed by the iQ5 optical System Software (Version 2.0). To normalize the data between the different biological samples, we calculated the ratio between the C_t_ value obtained for each GS primer pair and the housekeeping gene Elongation factor 1-α [42]. For the study of GS expression during nodule development the changes in expression for each time point analysed (3, 6, 10, 14 and 20 dpi) were calculated as fold change of the normalized C_t_ values relative to roots collected from nitrogen-starved plants just before *Rhizobium* inoculation.

### Protein extraction from plant tissues

Plant material was homogenized at 4°C in a mortar and pestle in extraction buffer (10 mM Tris pH 7.5, 5 mM sodium glutamate, 10 mM MgSO_4_, 1 mM DTT, 10% (v/v) glycerol and 0.05% (v/v) Triton X-100), the homogenates were centrifuged at 13 000 *g* for 20 min, at 4°C and the soluble fraction was recovered. Protein concentration was measured by the Coomassie dye-binding assay (Bio Rad) using BSA as a standard. For native gel electrophoresis, protein extracts were prepared as described above using an adapted GS extraction buffer (10 mM Tris pH 7.5, 5 mM sodium glutamate, 10 mM MgSO_4_, 0.5% digitonin). Following a first centrifugation at 13 000 *g* for 20 min, the extracts were ultracentrifuged at 100 000 g for 30 min at 4°C. Equal amounts of protein (30 μg) for each sample were first normalized to 20 μL and supplemented with 5 μl of Native-page loading dye (50% glycerol, 0.1% Ponceau S).

### Native PAGE electrophoresis and in-gel GS activity assays

Native electrophoresis was performed in NativePAGE™ Novex® 4-16% Bis-Tris Gels 1.0 mm (Invitrogen). The composition of anode buffer (25 mM imidazole/HCl, pH 7.0) and cathode buffer (0.05% Triton X-100, 50 mM Tricine, 7.5 mM imidazole, pH 7.0) were adapted from the method described by Wittig et al. [43]. Equal amounts (30 μg) of proteins, solubilized with digitonin, were loaded on each lane. Native gel electrophoresis was performed at 4°C under constant voltage (150 V) for 2–3 h. Following electrophoresis, the native gel was incubated in GS activity solution (0.2 mmol Tris pH 6.4, 0.2 mmol L-glutamine, 0.12 mmol Hydroxylamine, 1 μmol ADP, 2 μmol MnCl_2_ and 20 μmol sodium arsenate) at 30°C for 30 min. The reaction was stopped by adding a stop solution composed of 26% (w/v) FeCl_3_, 40% (w/v) Trichloroacetic acid and 3.3% (v/v) HCl. The brown colour characteristic of GS activity was detected in a few seconds and the gels were immediately digitalized.

### SDS-PAGE and western blot analysis

Soluble protein extracts were separated by 12.5% (w/v) SDS-polyacryamide gel electrophoresis (SDS-PAGE), in non-reducing conditions (using Laemmli’s sample buffer without β-mercaptoethanol), and electroblotted onto nitrocellulose membranes (Schleicher & Schuell).The proteins separated on Native-PAGE were electro-blotted onto PVDF membranes (GE Healthcare) using an electro-transfer buffer containing 10% methanol and 0.1% SDS. The membranes were incubated with primary antibodies: polyclonal GS antibody anti-Glnγ from *Phaseolus vulgaris*[27] or a rabbit polyclonal anti-peptide (SKSRTISKPVEHPSEL) antibody (Eurogentec), which specifically recognizes the plastid-located GS. Immuno-detection was performed using goat anti-rabbit peroxidase conjugated antibody (Vector Laboratories) and the ECL™ (GE healthcare, Lifesciences) detection system.

### Expression and purification of recombinant GS in *E. coli*

The coding sequences of *MtGS1a*, *MtGS1b*, and the sequences encoding the mature *MtGS2a* and *MtGS2b* polypeptides were amplified from the vector *pTrc99A*[44] using the vector primer 5′TTGACAATTAATCATCCGGC3′, and specific primers for *MtGS1a* (5′TGGTTGTGGTCGACTGGTTTCC3′); *MtGS1b* (5′AGCGTGGTGTCGACTGGTTTCC3′); *MtGS2a* (5′AATAGATGTCGACTTTCAATGC 3′) and *MtGS2b* (5′AATAGATGTCGACCTTCAATGC3′). The amplified fragments were digested with *Nco*I and *Sal*I and cloned into the *Nco*I and *Xho*I sites of *pET-24-d-T* vector, a derivative of *pET-24-d (+)* (Novagen), containing a C-terminal His-tag. The resulting plasmids encode C-terminal His_6_-tagged GS fusion proteins. The constructs were sequenced to ensure no mistakes have been introduced.

To express the His-tagged GS proteins, *Escherichia coli* BL21 codon^+^ (DE3) RIL cells (Stratagene) harbouring the plasmids pET-24d-GS were first cultured on LB medium at 37°C to mid-exponential growth (OD_600_ = 0.5). The expression of the recombinant proteins was then induced by IPTG (final concentration 1 mM) and the cell growth proceeded overnight at 20°C. The cells were harvested by centrifugation at 2800 g, resuspended in extraction buffer (10 mM Hepes pH 7.4, 10 mM MgSO_4_, 5 mM sodium glutamate, 500 mM NaCl, 20 mM imidazole), disrupted by sonication, and centrifuged (60 minutes, 38000 g, 4°C) to remove cell debris. The crude protein extract was filtered through a 5 μm low protein-binding filter, and loaded onto a 5 mL Ni-Sepharose column (GE Healthcare) equilibrated in buffer A (10 mM Hepes pH 7.4, 500 mM NaCl, 20 mM imidazole). Elution of the bound fusion proteins was achieved by increasing the buffer A imidazole concentration to 230 mM. The GS-containing fractions were pooled and dialyzed against 10 mM Hepes pH 7.4 and concentrated to 5 mg.mL^-1^ on a centrifugal concentration device with a 10 kDa MWCO membrane (VIVASCIENCE).

### Evaluation of GS activity and kinetics

GS activity was determined by quantification of γ-glutamyl hydroxamate produced either by the transferase (GSt) or the synthetase (GSs) reactions as previously described by Culimore and Sims [45]. γ-glutamyl hydroxamate concentration was determined spectrophotometrically (Ultrospec 1000 UV/Visible, Pharmacia) at 500 nm. Reactions started by addition of protein extracts or purified His-tagged GS isoenzymes to an assay mixture composed of 80 mM Tris pH 6.4, 100 mM glutamine, 0.04 mM ADP, 60 mM hydroxylamine, 1 mM MnCl_2_ and 30 mM sodium arsenate for the transferase assay, and 100 mM Tris pH 7.8, 100 mM glutamate, 8 mM ATP, 8 mM Hydroxylamine and 16 mM MgSO_4_ for the synthetase assay. The reaction proceeded at 30°C for 2 min (transferase) or 5 minutes (synthetase) and was stopped by addition of a solution composed of 26% (w/v) FeCl_3_, 40% (w/v) Trichloroacetic acid and 3.3% (v/v) HCl. GS specific activity was determined as μmol of γ-glutamyl hydroxamate produced per minute per mg of protein. The kinetic properties of the purified GS isoenzymes were determined using the synthetase reaction. Assays were performed in 96-well plates (uQuant microplet spec, Biotek), at 30°C, using the same amount of protein 10 μg (GS1a; GS2a and GS2b) or 20 μg (GS1b) and varying concentrations of each substrate in the assay mixture. The amount of γ-glutamyl hydroxamate produced was considered to increase linearly during the first three minutes for all four isoenzymes. To determine the kinetic properties of the recombinant isoenzymes, first, the “initial rate” (*v*_*0*_; μmol.min^-1^) for each reaction was estimated as the slope (constrained to pass through the origin) of the linear function γ-glutamyl hydroxamate = f (time). Secondly, the dependence of *v*_*0*_ on substrate concentration ([S]) was evaluated. The catalytic properties *V*_*max*_ (maximum velocity; μmol.min^-1^), *K*_*m*_ (Michaelis-Menten constant; mM), *K*_*0.5*_ (Half-saturation constant; mM), *k*_*cat*_ (turnover number; s^-1^) were automatically calculated by non-linear regression data analysis using the Prism5® software (GraphPad software Inc.). Two equations were used to calculate the catalytic properties, one assuming a hyperbolic dependence of *v*_*0*_ on [S] (Michaelis-Menten equation) and the other assuming a sigmoidal dependence (allosteric sigmoidal equation). The pH optimum was determined using the standard synthetase reaction mixture with Tris buffer of varying pH (6.0, 6.5, 7.0, 7.5, 8.0, 8.5).

## Abbreviations

GS: Glutamine Synthetase; NUE: Nitrogen use efficiency; qRT-PCR: Quantitative real time polymerase chain reaction; SDS-PAGE: Sodium dodecyl sulphate polyacrylamide gel electrophoresis.

## Authors’ contributions

AS carried out most of the experimental work, participated in the design of the study and helped to draft the manuscript. LS carried out part of the experimental work. HC conceived and coordinated the study and drafted the manuscript. All authors read and approved the final manuscript.
